# Polarization Raman spectra of graphene driven by monolayer ReS_2_

**DOI:** 10.1515/nanoph-2025-0286

**Published:** 2025-10-16

**Authors:** Xiangtai Xi, Haoran Liu, Chengxiang Gou, Zeyu Sun, Xilin Zhou, Xin Xie, Jinping Li, Zhenglong Zhang, Hairong Zheng, Hongxing Xu

**Affiliations:** School of Physics and Information Technology, 12401 Shaanxi Normal University, Xi’an 710062, China; Stage Key Laboratory of Precision Spectroscopy, East China Normal University, Shanghai 200241, China; School of Physics and Technology, Wuhan University, Wuhan 430072, China

**Keywords:** graphene, ReS_2_, two dimensional materials, Van der Waals heterojunction, polarization Raman spectroscopy

## Abstract

Precise control of the polarization state of light at the nanoscale is a critical and transformative technology for advancing next-generation nano-optical components. Graphene, despite its highly symmetric lattice and weak in-plane anisotropy, exhibits limited optical response to polarized light in the visible spectrum. This constraint significantly hinders its application in visible-light polarization detection and broader optoelectronic integration. In contrast, two-dimensional (2D) layered rhenium disulfide (ReS_2_), characterized by its unique distorted 1T-phase crystal structure, exhibits strong in-plane anisotropy in the visible range. This intrinsic property not only enriches its optical characteristics but also substantially enhances the photocurrent and photoresponsivity of graphene when integrated, thereby offering new opportunities for advanced optoelectronic applications. In this study, we fabricated a vertically stacked graphene/ReS_2_ van der Waals heterostructure with high precision. Polarized Raman spectroscopy was employed to systematically analyze the polarization dependence of the heterojunction, enabling an in-depth understanding of its structural and optical anisotropy. Our experimental results reveal that when isotropic single-layer graphene is coupled with anisotropic ReS_2_, the resulting heterostructure exhibits anomalous polarization-dependent Raman scattering. This finding highlights the potential of ultrathin 2D heterostructures as nanoscale polarization-sensitive optical elements. This work not only provides a novel approach for designing and implementing next-generation polarization-resolved optical devices based on 2D anisotropic materials but also lays the groundwork for their integration into highly miniaturized and lightweight optoelectronic systems. These findings hold significant promise for advancing the field of optoelectronics and enabling the development of more sophisticated and efficient optical technologies.

## 1 Introduction

The development of integrated nano-optical and optoelectronic devices necessitates efficient strategies for modulating optical responses at the nanoscale [1], [2], [3]. Recent advances in this field have highlighted the use of plasmonic nanostructured resonators and ultrathin dielectric materials [4], [5], [6]. Among these, 2D layered crystals have garnered significant attention for their exceptional photonic properties, making them ideal candidates for miniaturized optical systems [7], [8]. The optical behavior of 2D layered crystals is primarily governed by their strong light–matter interactions, which allow for precise modulation of light phase and intensity [9]. Graphene, for instance, has been successfully employed in broadband transverse flux applications and optically transparent microwave polarizers [10]. It has also been utilized in the fabrication of atomically thin optical lenses and diffraction gratings [11], [12]. However, due to its in-plane isotropic nature, graphene’s capacity to manipulate polarized light relies mainly on the differential interaction of light polarized parallel versus perpendicular to the plane of the 2D material [13]. This inherent limitation underscores the importance of exploring anisotropic 2D materials for advanced polarization-dependent optical functionalities [14].

In recent years, ReS_2_, a 2D material exhibiting in-plane grating-like anisotropy, has garnered significant attention [15]. Due to its distorted 1T-phase lattice structure, monolayer ReS_2_ displays pronounced in-plane optical anisotropy, distinguishing it from graphene and many other 2D materials [16]. Few-layer ReS_2_ further demonstrates strong linear dichroism and birefringence [17]. Although ReS_2_ has been proposed as a promising candidate for linear optical polarizers, most previous studies have primarily focused on its intrinsic optical and optoelectronic properties or its application in pn junctions – for example, in the development of broadband polarization-sensitive photodetectors [18]. However, the potential of ReS_2_ to actively modulate the polarization state of light through interaction with a secondary material remains largely unexplored [19]. Its capability to influence the optical response of heterostructures or composite systems has yet to be conclusively demonstrated.

In this study, we fabricated vertically stacked graphene/ReS_2_ heterostructures and performed angle-resolved polarized Raman spectroscopy to investigate their optical behavior. The results reveal that the 2D Raman peaks of graphene within the heterostructure exhibit polarization-dependent characteristics. Notably, the direction of maximum Raman scattering intensity for the graphene 2D peak is orthogonal to the polarization direction of the MODIII vibrational mode in ReS_2_. These findings demonstrate that ReS_2_ not only induces anomalous polarization-dependent Raman scattering but also acts as a functional optical anisotropic medium capable of modulating the optical response of graphene. This work provides a promising strategy for designing novel micro-optical components based on two-dimensional anisotropic materials and offers a practical route for their seamless integration into nanophotonic and optoelectronic devices.

## 2 Experiment

**Figure 1:**
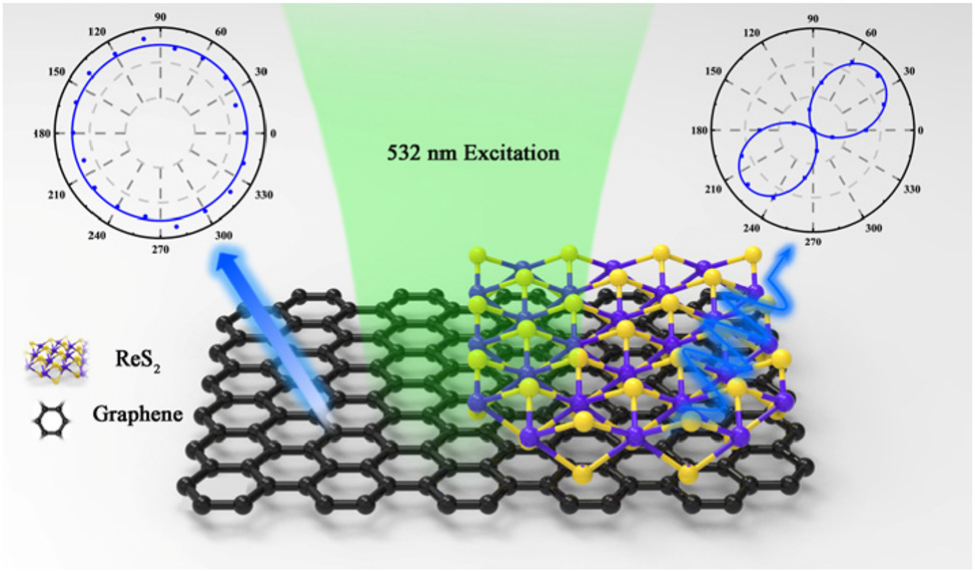
Schematic diagram of the experimental configuration.

Monolayer ReS_2_ was synthesized on atomically flat mica substrates via chemical vapor deposition (CVD) [20]. Subsequently, a vertical van der Waals heterostructure was assembled by transferring graphene onto the ReS_2_ layer using a wet chemical transfer method, followed by vacuum annealing to improve interface quality. Optical microscopy was employed to image both the graphene and ReS_2_ sheets, while their thicknesses were precisely characterized using atomic force microscopy (AFM). Raman scattering measurements were performed using a RENISHAW inVia Reflex™ Raman system. For optical imaging, a halogen lamp was used as the white light source. A polarizer was placed in the incident light path to adjust the polarization direction of the excitation beam. In the angle-resolved polarized Raman experiments, a 532 nm laser was used to excite the Raman signals. The principal angular-resolved Raman data were acquired in the parallel-polarized configuration, i.e., the linear polarization of the incident beam and the analyzed polarization of the collected Raman signal were kept parallel for each angular step. During the measurement, a 60× objective lens was used, with a 532 nm excitation light source, and the output power of the objective lens was 1 mw. The acquisition times were set to 2 acc, and the spectral acquisition time was 5 s. During the first acquisition, half of the polarizer was adjusted to the 0 mark. After the first acquisition, the half-wave plate was rotated so that its reading was collected every 10°, meaning that the electric field direction of the laser from the objective lens changed by 20° each time. The rotation range of the half-wave plate was 0°–90°, and the rotation range of the excitation optical vector was 0°–180°. The measured results were then analyzed and processed. Figure 1 schematically illustrates the system studied in this work, with the aim of comparing and analyzing the differences between the van der Waals heterostructure and the region without ReS_2_ coverage.

## 3 Results and discussion

**Figure 2:**
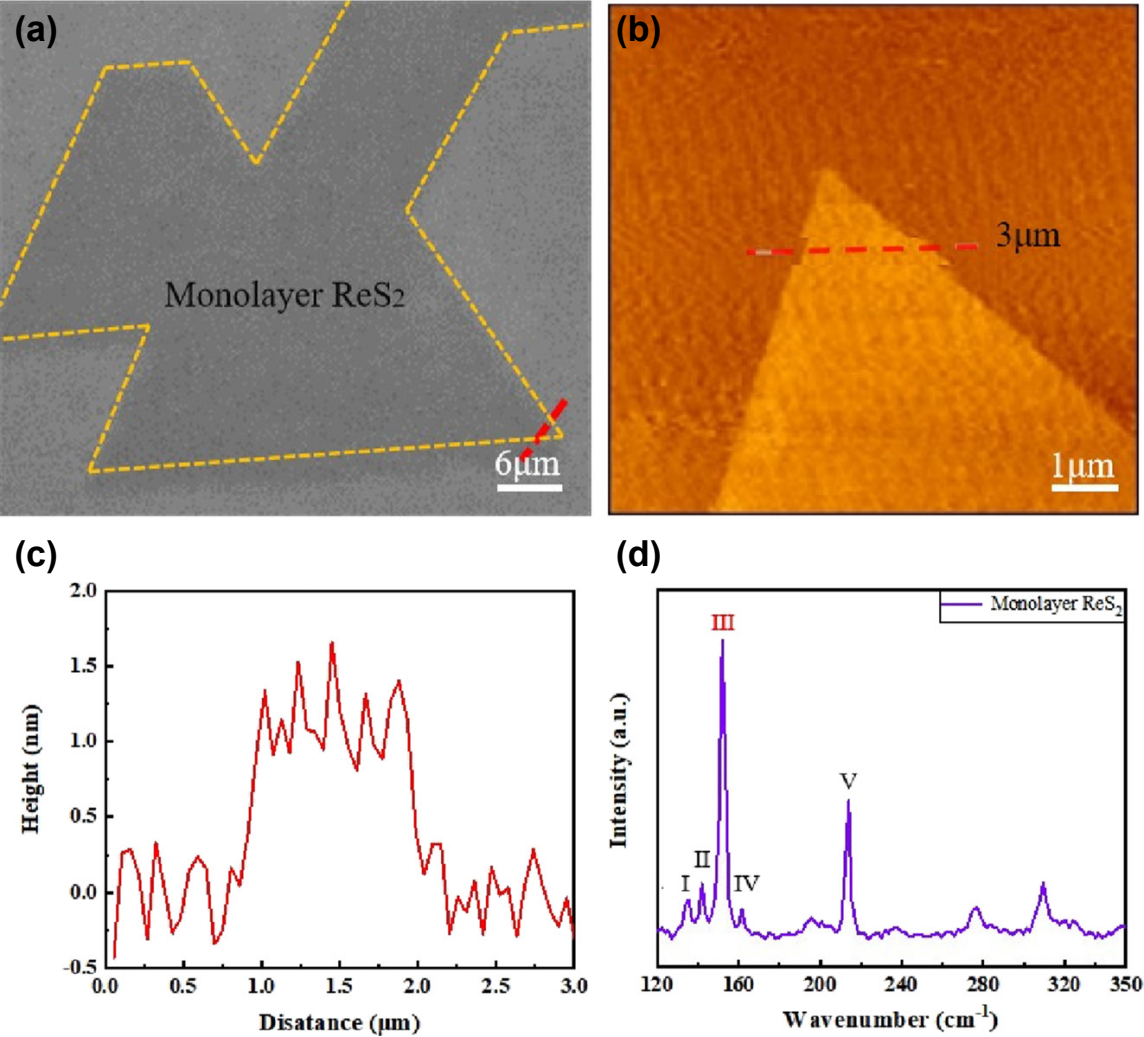
Structural characterization of monolayer ReS_2_. (a) Optical image of single-layer ReS_2_. The orange dotted area represents single-layer ReS_2_. (b) AFM image of single-layer ReS_2_. (c) Height variation curve obtained along the position of the blue solid line in (b). (d) The Raman scattering spectrum of single-layer ReS_2._

In Figure 2(a), the optical image of the monolayer ReS_2_ is outlined by a yellow dotted line. To determine the layer thickness, atomic force microscopy (AFM) measurements were conducted along the red dotted line indicated in Figure 2(a). The corresponding height profile is shown in Figure 2(b), revealing a measured thickness of approximately 0.9 nm, consistent with previously reported values for monolayer ReS_2_ [21]. Figure 2(c) presents the height variation along the red dotted line in Figure 2(b), confirming the uniform thickness characteristic of monolayer materials. Figure 2(d) displays the unpolarized Raman spectrum of the ReS_2_ monolayer. ReS_2_ has been reported to exhibit up to sixteen Raman-active vibrational modes due to its low-symmetry triclinic structure; in this work, we focus on a subset of these modes and highlight only those vibrational patterns most relevant to our experimental objectives. According to previous studies on ReS_2_, which shares a similar distorted crystal structure, a total of 18 Raman-active modes are expected. In this study, we focus on the spectral range of 120–240 cm^−1^, as the most prominent vibrational modes appear within this window. For simplicity, the observed Raman modes are labeled in ascending order of wavenumber as Modes I through V, with approximate peak positions at 135 cm^−1^, 141 cm^−1^, 150 cm^−1^, 160 cm^−1^, and 211 cm^−1^, respectively. Among them, Mode III (MODIII) is primarily used for subsequent analysis.

**Figure 3:**
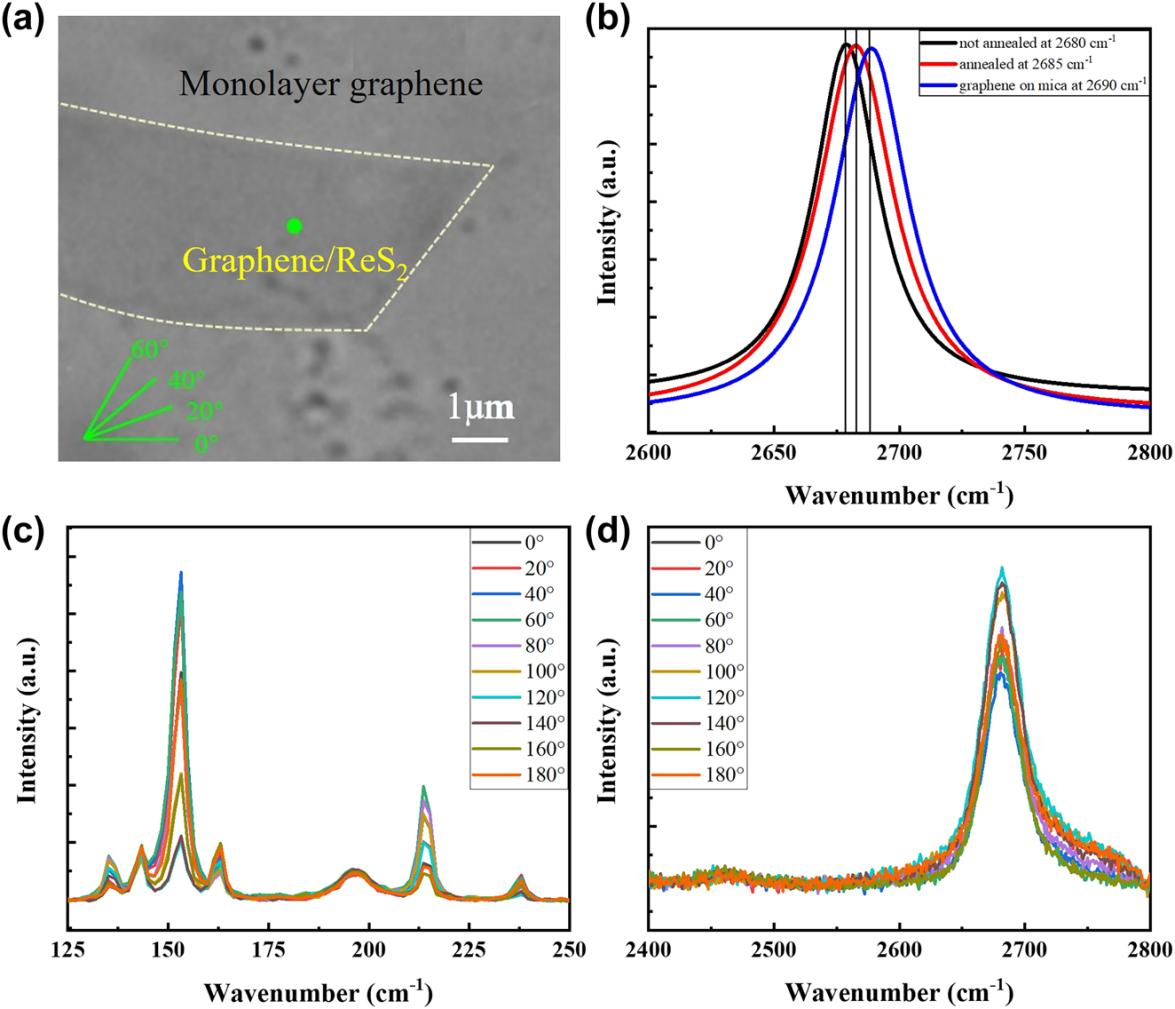
Characterization of heterogeneous structural domains and polarized Raman spectroscopy measurements. (a) Optical image of graphene/ReS_2_. The illustration in the lower left corner shows the direction of the polarized photoelectric vector. (b) Comparison of two-dimensional peaks of monolayer graphene, unannealed heterostructures and annealed heterostructures on mica substrates, with data normalization. (c) and (d) Are the polarized Raman spectra of ReS_2_ and graphene 2D peaks at the green sampling point in Figure (a).

We employed a wet chemical transfer method to transfer monolayer graphene, initially supported on a copper substrate, onto monolayer rhenium disulfide (ReS_2_). Following drying and annealing treatments, a vertically stacked van der Waals heterostructure was successfully fabricated. An optical image of the sample is shown in Figure 3(a), where the heterostructure region is delineated by a pale-yellow dotted line. The direction of the excitation photoelectric vector is indicated by the inset in the lower-left corner. It is well established that the Raman peaks of graphene are sensitive to external influences such as strain, temperature, and charge transfer [22]. Therefore, Raman spectroscopy was employed to characterize monolayer graphene on mica substrates, as well as graphene in both unannealed and annealed heterostructures. All Raman spectra were normalized for comparison. As shown in Figure 3(b), the 2D peaks of graphene for the three different samples are represented by blue, black, and red curves, respectively. For graphene on the mica substrate, the 2D peak appears at approximately 2,690 cm^−1^. This blue shift is attributed to electron transfer from the mica substrate to the graphene, which affects the electron–phonon coupling. In contrast, the 2D peak of graphene in the unannealed heterostructure is located near 2,680 cm^−1^. This is likely due to the presence of a thin air gap between the graphene and ReS_2_ layers prior to annealing, which inhibits strong interfacial interaction and thus results in minimal shift in the Raman peak.

Upon annealing, the Raman 2D peak of graphene within the heterojunction region exhibited a notable blue shift, with its position shifting to approximately 2,685 cm^−1^. This spectral shift provides clear evidence of enhanced interlayer interaction between graphene and ReS_2_. Under 532 nm laser excitation, electrons in both ReS_2_ and graphene are promoted from the valence band to the conduction band. Due to the mismatch in their electronic band structures, some holes in graphene are transferred to the valence band of ReS_2_, while a portion of the electrons in the conduction band of ReS_2_ migrate into the conduction band of graphene. This charge transfer process modifies the carrier density in graphene and results in the observed blue shift of its Raman 2D peak, consistent with previous literature reports [19]. The angle-resolved Raman spectra of ReS_2_ are shown in Figure 3(c), with the spectral window adjusted to 125–250 cm^−1^ to better highlight its in-plane anisotropy. The MODIII mode exhibits pronounced polarization dependence, clearly revealing the material’s anisotropic vibrational characteristics. As shown in Figure 3(d), the maximum Raman intensity of the graphene 2D peak occurs at an incident polarization angle of approximately 120°, further confirming the polarization-dependent nature of the interfacial interaction.

**Figure 4:**
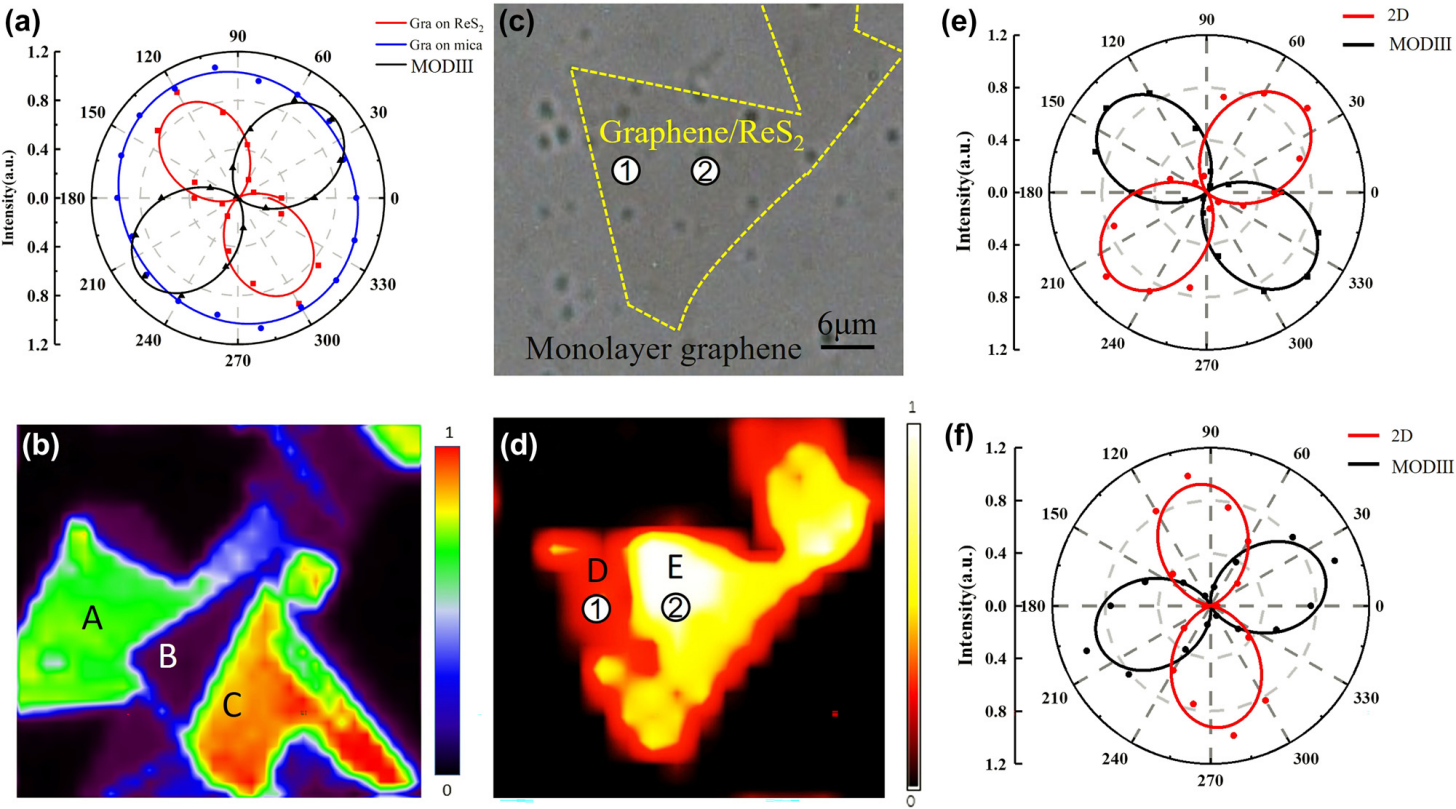
Anisotropic optical interactions arising from interlayer coupling in 2D heterojunctions (a) polarized Raman spectra of graphene and ReS_2_ in heterogeneous structures. Among them, the black solid line represents the polarized Raman spectrum of ReS_2_ in the heterogeneous structure, while the blue solid line and the red solid line correspond respectively to graphene on the mica substrate and graphene in the heterogeneous structure. (b) The mapping spectrum of single-layer ReS_2_ 211 cm^−1^ peak position. (c) Optical image of the heterostructure. (d) Plotting the peaks of ReS_2_ 213 cm^−1^ in heterogeneous structures. (e) Polarized Raman spectra of graphene and ReS_2_ at sampling point 1. (f) Polarized Raman spectra of graphene and ReS_2_ at sampling point 2.

Angle-resolved polarized Raman spectra were obtained by extracting the 2D peaks of graphene in the heterostructure and in monolayer graphene on mica substrates, as illustrated in Figure 4(a). Additionally, the MODIII vibrational mode of ReS_2_ was extracted from the heterostructure and normalized, shown as the black curve in Figure 4(a). The blue curve represents the 2D Raman peak of monolayer graphene on mica, while the red curve corresponds to graphene within the heterostructure. As evident from the figure, monolayer graphene on the mica substrate displays in-plane isotropy, producing a nearly perfect circular pattern in the polarization plot. In contrast, due to interlayer interactions with ReS_2_, the graphene within the heterostructure exhibits clear in-plane anisotropy. The polarization directions corresponding to the maximum and minimum intensities of the graphene 2D peak are approximately 130° and 40°, respectively. Moreover, the MODIII vibrational mode of ReS_2_ shows its maximum and minimum polarization intensities at 40° and 130°, respectively – orthogonal to those of the graphene 2D peak. Notably, when the polarization angle of the incident electric field is rotated counterclockwise from 40° to 130°, the Raman intensity of the ReS_2_ MODIII mode increases, while the 2D peak intensity of graphene decreases, indicating an inverse relationship between their polarization-dependent responses. This behavior strongly suggests a coupled anisotropic optical interaction between the two layers.

In free-standing graphene, the in-plane G mode originates from the doubly-degenerate E_2_g representation, which in the high-symmetry limit leads to an isotropic Raman tensor [23]. When graphene is placed on an in-plane–anisotropic substrate such as ReS_2_, the interface perturbation lowers the effective symmetry and can split the degeneracy of the E_2_g mode [24]. Consequently, the effective Raman tensor acquires anisotropic elements, resulting in a polarization-dependent Raman intensity that depends on the relative angle between the light polarization and the ReS_2_ domain orientation. Here are some mechanisms that can generate the observed anisotropy. Interface-induced anisotropic strain: Anisotropic strain in graphene, induced by epitaxial alignment or transfer on ReS_2_, can split the G mode and change its polarization response [24]. Local-field effects: ReS_2_ has in-plane anisotropic optical response; the local field experienced by graphene is thus polarization dependent. Anisotropic dielectric/local-field effects arise because ReS_2_ exhibits strong in-plane linear dichroism; this modifies the local optical field and hence the effective Raman tensor of graphene [25].

The in-plane vibrational peak of rhenium atoms at 211 cm^−1^ was identified from the Raman spectra of the samples, and a corresponding Raman mapping was generated, as shown in Figure 4(b). In this map, the high-quality monolayer ReS_2_ is distinctly divided into three large, relatively regular domains, along with several smaller regions. These three major domains are labeled as Region A, Region B, and Region C. When the linearly polarized laser is incident at 0°, Region C exhibits the highest Raman peak intensity, represented by the red area, whereas Region B shows the weakest signal intensity, indicated by dark purple. Furthermore, the Raman intensities within each of these domains are uniformly distributed. These observations suggest that the different regions correspond to distinct sub-crystal domains, each characterized by an excellent and uniform lattice structure, thereby providing a robust foundation for subsequent Raman spectroscopy measurements.

To more effectively elucidate the interaction between ReS_2_ and graphene within the heterostructures, large-area vertical heterostructures were fabricated again. Figure 4(c) shows the optical image of the heterostructure, where the region enclosed by the light yellow dotted box corresponds to the heterostructure area, and the region outside the box represents the monolayer graphene area. To distinguish the different subdomains of ReS_2_ within the heterostructure, Raman spectroscopy mapping was performed over the entire heterostructure region. The Raman peak positions of ReS_2_ at 213 cm^−1^ were extracted from the spectra to generate the mapping image shown in Figure 4(d). This image clearly reveals two large ReS_2_ subdomains within the heterostructure, labeled as subdomains D and E. Subsequently, representative measurement points were selected from the Raman mapping, and polarization-resolved Raman spectra were acquired at these locations. Specifically, sampling points 1 and 2 were chosen within the D and E subdomains, respectively, for angle-resolved polarized Raman spectroscopy measurements.

At sampling point 1, the angle-resolved polarized Raman intensity spectra of the MODIII vibration mode of ReS_2_ and the two-dimensional (2D) peaks of graphene were extracted, as shown in Figure 4(e). After normalization, the data reveal that within subdomain D, the maximum Raman intensity of the ReS_2_ MODIII mode occurs at an incident polarization angle of 140°, while the minimum is observed at 50°. As the polarization angle increases from 50° to 140°, the Raman intensity of ReS_2_ gradually increases. In contrast, the 2D Raman peak intensity of graphene exhibits an opposite trend: it reaches its maximum at 50° and its minimum at 140°. Thus, the polarization-dependent Raman response of graphene displays an inverse angular dependence relative to the MODIII vibration mode of ReS_2_. This anti-correlated behavior highlights the interlayer coupling and anisotropic optical interaction within the heterostructure.

The angle-resolved polarization Raman intensity spectrum at sampling point 2 is presented in Figure 4(f). After normalization, the data show that the maximum intensity of the ReS_2_ MODIII vibration mode occurs at a polarization angle of 20°, while the minimum appears at 110°. As the angle increases from 20° to 110°, the Raman intensity of ReS_2_ gradually decreases. In contrast, the two-dimensional (2D) Raman peak of graphene reaches its maximum at 110° and its minimum at 20°, displaying an inverse angular dependence relative to the ReS_2_ mode. Between 60° and 150°, the Raman intensity of graphene progressively increases. These results are consistent with previous observations. By comparing the polarization directions of the maximum 2D peak intensities in Figure 4(e) and (f), a maximum angular deviation of 60° is observed, matching the shift in the polarization direction of the MODIII vibration mode. This phenomenon suggests a correlation between interlayer charge transfer within the heterostructure and the strong linear dichroism of ReS_2_. Charge transfer modifies the band structure of graphene, while the pronounced dichroism of ReS_2_ can modulate the polarization state of incident light, thereby inducing anomalously polarized Raman scattering in graphene. However, the underlying physical mechanism remains incompletely understood and warrants further investigation.

## 4 Conclusions

In summary, by exploiting the two-dimensional nature and pronounced in-plane optical anisotropy of ReS_2_, we constructed a graphene/ReS_2_ vertical heterostructure that exhibits angle-dependent anomalous Raman scattering in graphene. Angle-resolved polarized Raman spectroscopy revealed that ReS_2_ can induce polarization-dependent Raman responses in graphene, enabling the latter to display a pronounced optical anisotropy under visible-light excitation. Notably, the direction of maximum Raman intensity in the 2D peak of graphene was found to be perpendicular to the polarization direction of the MODIII vibrational mode of ReS_2_. Furthermore, the polarization behavior of graphene can be modulated by adjusting the orientation of the ReS_2_ rhenium chains within the heterostructure. These findings highlight a promising strategy for designing ultrathin optical polarizers based on two-dimensional anisotropic crystals. Such structures offer a practical and integrable solution for polarization control in nanophotonic and optoelectronic devices, holding significant potential for future applications in advanced photonic systems.

## References

[1] Sun Z., Martine A., Wang F. (2016). Optical modulators with 2D layered materials. *Nat. Photonics*.

[2] Li Y., Qian F., Xiang J., Lieber C. M. (2006). Nanowire electronic and optoelectronic devices. *Mater. Today*.

[3] Koppens F. H., Mueller T., Avouris P., Ferrari A. C., Vitiello M. S., Polini M. (2014). Photodetectors based on graphene, other two-dimensional materials and hybrid systems. *Nat. Nanotechnol.*.

[4] Shakoor A., Grant J., Grande M., Cumming D. R. S. (2019). Towards portable nanophotonic sensors. *Sensors*.

[5] Zhao Y., Belkin M. A., Alu A. (2012). Twisted optical metamaterials for planarized ultrathin broadband circular polarizers. *Nat. Commun.*.

[6] Wei H., Xu H. (2012). Nanowire-based plasmonic waveguides and devices for integrated nanophotonic circuits. *Nanophotonics*.

[7] Zhang H. (2015). Ultrathin two-dimensional nanomaterials. *ACS Publ.*.

[8] Wang Q. H., Kalanter-Zadeh K., Kis A., Coleman J. N., Strano M. S. (2012). Electronics and optoelectronics of two-dimensional transition metal dichalcogenides. *Nat. Nanotechnol.*.

[9] Xu M., Liang T., Shi M., Chen H. (2013). Graphene-like two-dimensional materials. *Chem. Rev.*.

[10] Yan D., Li E., Feng Q., Li X., Guo S. (2022). Design and analysis of a wideband microwave absorber based on graphene-assisted metamaterial. *Optik*.

[11] Grande M. (2015). Optically transparent microwave polarizer based on quasi-metallic graphene. *Sci. Rep.*.

[12] Kong X. T. (2015). Graphene-based ultrathin flat lenses. *ACS Photonics*.

[13] Lin L. (2019). Towards super-clean graphene. *Nat. Commun.*.

[14] Malard L. M., Pimenta M. A., Dresselhaus G., Dresselhaus M. (2009). Raman spectroscopy in graphene. *Phys. Rep.*.

[15] Li X. (2019). Nanoassembly growth model for subdomain and grain boundary formation in 1T′ layered ReS_2_. *Adv. Funct. Mater.*.

[16] Chenet D. A. (2015). In-Plane anisotropy in Mono- and few-layer ReS_2_ probed by raman spectroscopy and scanning transmission electron microscopy. *Nano Lett.*.

[17] Gehlmanm M. (2017). Direct observation of the band gap transition in atomically thin ReS_2_. *Nano Lett.*.

[18] Gong C. (2017). Electronic and optoelectronic applications based on 2D novel anisotropic transition metal dichalcogenides. *Adv. Sci.*.

[19] Liu M. (2019). Monolayer-ReS_2_ field effect transistor using monolayer-graphene as electrodes. *Phys. B: Condens. Matter*.

[20] He X. (2015). Chemical vapor deposition of high-quality and atomically layered ReS_2_. *Small*.

[21] Liu F. (2016). Highly sensitive detection of polarized light using anisotropic 2D ReS_2_. *Adv. Funct. Mater.*.

[22] Wu J., Xu H., Zhang J. (2014). Raman spectroscopy of graphene. *Acta Chimica Sinica*.

[23] Ferrari A. C., Basko D. M. (2013). Raman spectroscopy as a versatile tool for studying the properties of graphene. *Nat. Nanotechnol.*.

[24] Yoon D., Son Y. W., Cheong H. (2011). Strain-dependent splitting of the double-resonance raman scattering band in graphene. *Phys. Rev. Lett.*.

[25] Tongay S. (2014). Monolayer behaviour in bulk ReS2 due to electronic and vibrational decoupling. *Nat. Commun.*.

